# The functional genomics laboratory: functional validation of genetic variants

**DOI:** 10.1007/s10545-018-0146-7

**Published:** 2018-02-14

**Authors:** Richard J. Rodenburg

**Affiliations:** Radboudumc, Radboud Center for Mitochondrial Medicine, 774 Translational Metabolic Laboratory, Department of Pediatrics, PO Box 9101, 6500HB Nijmegen, The Netherlands

## Abstract

Currently, one of the main challenges in human molecular genetics is the interpretation of rare genetic variants of unknown clinical significance. A conclusive diagnosis is of importance for the patient to obtain certainty about the cause of the disease, for the clinician to be able to provide optimal care to the patient and to predict the disease course, and for the clinical geneticist for genetic counseling of the patient and family members. Conclusive evidence for pathogenicity of genetic variants is therefore crucial. This review gives an introduction to the problem of the interpretation of genetic variants of unknown clinical significance in view of the recent advances in genetic screening, and gives an overview of the possibilities for functional tests that can be performed to answer questions about the function of genes and the functional consequences of genetic variants (“functional genomics”) in the field of inborn errors of metabolism (IEM), including several examples of functional genomics studies of mitochondrial disorders and several other IEM.

## Whole exome and whole genome sequencing

The introduction of next generation sequencing, and in particular of whole exome sequencing (WES), as a tool in a routine molecular genetics diagnostics setting, has revolutionized the field of molecular genetics and IEM. In the pre-NGS era, often a large number of functional diagnostic tests, such as metabolite screening and enzyme analysis, were needed to establish a biochemical diagnosis and to identify candidate genes, which were subsequently sequenced one by one. Nowadays, WES is used routinely as a tool to diagnose many different IEMs. WES is particularly suitable to diagnose disorders with a large number of candidate genes and a broad clinical spectrum, such as mitochondrial disorders or congenital disorders of glycosylation (Timal et al. 2012; Wortmann et al. 2015, 2015). In addition, both the cost-effectiveness and quality of the sequence data have increased tremendously in the past few years, which has made WES also a suitable tool to test patients for metabolic genetic disorders with a more defined clinical spectrum and smaller number of candidate genes, including peroxisomal disorders, fatty acid oxidation disorders, and many more (Lines et al. 2014; Haack et al. 2015).

The reduction in costs, the relatively simple sample collection procedure, and the broad genetic screening potential of WES are the main reasons for the current trend of applying WES at earlier stages of the diagnostic work-up of patients than before. In cases where this approach results in the identification of a clear genetic defect, e.g., a known pathogenic mutation in a disease gene associated with a phenotype that is the same as that of the patient under investigation, the benefits of such an “exome-first” approach are obvious. However, in spite of the tremendous progress that has been made in the development of both sequencing technology and bioinformatics in recent years, it should be noted that in the majority of investigations, whole exome (or genome) sequencing does not result in a genetic diagnosis (Neveling et al. 2013), and in those cases other diagnostic tests are still necessary to confirm or reject a diagnosis with certainty. In this paper, the problem of genetic variants of unknown clinical significance is explained, focusing on the field of IEM, and an overview is given of strategies and methods to study these genetic variants in order to understand the functional consequences of genetic variants (“functional genomics”). At the level of the individual patient, the functional data may provide key evidence to change a possible diagnosis into a certain diagnosis. These functional studies can consist of specific tests to investigate a certain genetic variant of unknown clinical significance identified in an individual patient or family. In addition, this paper also describes some examples of prospective studies in which functional data is collected to generate sequence-function maps of genes. Such data can serve as reference data for the interpretation of genetic variants that will be identified in molecular diagnostics tests in the future. Both approaches will ultimately lead to an understanding of the function of the entire genome and its variants.

## Outcomes of WES/WGS

**Fig. 1:**
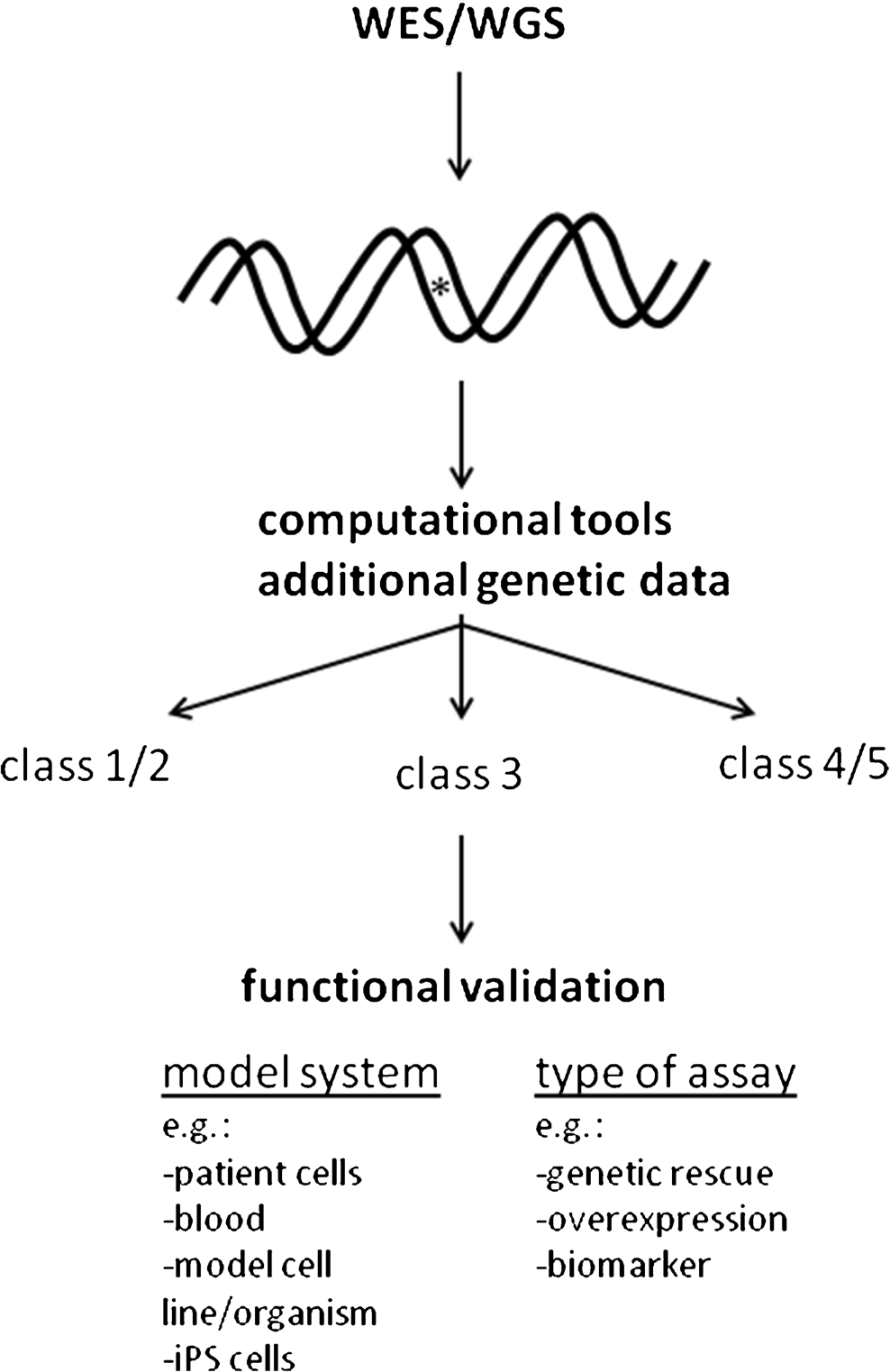
From genetic test to functional validation. After whole exome (or genome) sequencing, genetic variants are analyzed by bioinformatics tools and additional genetic tests (e.g., segregation analysis, population studies) are performed. On the basis of these data, variants are classified as not/unlikely pathogenic (class 1/2), of unknown pathogenicity (class 3), or likely/definite pathogenic (class 4/5). For class 3 variants, functional validation studies are a powerful tool to obtain evidence for possible pathogenicity

**Fig. 2:**
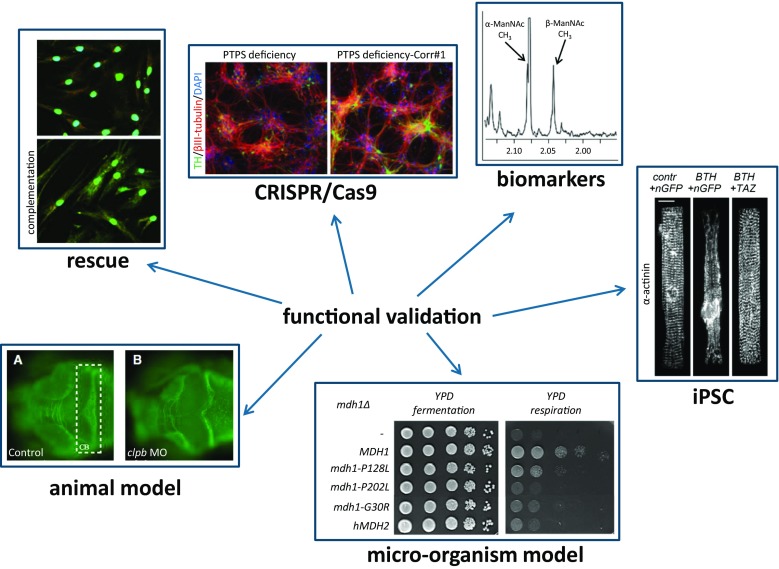
Examples of functional genomics approaches. These approaches, or combinations thereof, are frequently used to investigate the pathogenicity of genetic variants of unknown clinical significance. The examples shown are described in a clockwise order starting in the top-left corner. Rescue: introduction by lentiviral transduction of wild type *LYRM7* cDNA in fibroblasts from a patient with a defect in *LYRM7* results in normalization of mitochondrial Rieske Fe-S protein (Hempel et al. 2017). CRISPR/Cas9: Absence of thymidine hydroxylase (TH) staining in iPSC-derived dopaminergic nerve cells from a patient with a defect in *PTPS*, and normalization of TH expression after CRISPR/Cas9-mediated correction of the *PTPS* gene (Ishikawa et al. 2016). Biomarkers: detail of a 1H NMR spectrum of a CSF sample from a *NANS* patient, showing the presence of alpha and beta forms of *N*-acetylmannosamine (van Karnebeek et al. 2016). iPSC: Abnormal sarcomere organization in iPSC-cardiomyocytes derived from fibroblasts from a Barth-syndrome patient (BTH) with mutations in the tafazzin gene (*TAZ*), and normalization of sarcomeres after transfection with *TAZ*-mRNA (Wang et al. 2014). Micro-organism model: Restoration of growth on a non-fermentable carbon source (YPG) of a mitochondrial malate dehydrogenase-deficient yeast strain (*mdh1Δ*) by transfection with wild type *MDH1*, but not with various *mdh*-mutants (Ait-El-Mkadem et al. 2017). Animal model: A cerebellar defect in *CLPB* knock-down zebrafish embryos, as seen in Ac-tubulin stained embryos (the cerebellum is indicated by the rectangle in the control animal) (Wortmann et al. 2015, 2015)

There are different approaches to WES data analysis. For several reasons, in Nijmegen we have adopted a two step approach. In step 1, the WES data is filtered using a virtual gene panel of clinically relevant genes based on the clinical phenotype of the patient under investigation (Neveling et al. 2013). This minimizes the chance of unsolicited findings, and focuses the data analysis on relevant candidate genes only, which increases the chance of identifying disease causing variants in the WES data. If negative, an optional second step is an exome-wide investigation of the WES data outside the virtual gene panel (or panels) investigated in step 1. The success of WES depends not only on the technical quality of the sequencing data, but also on the amount of clinical- and other patient-derived data that is available. The more specific clinical information is known, the better the quality of the interpretation of the WES data, and the smaller the chance of unsolicited genetic findings. For this reason, several tools have been developed to combine exome sequencing data with clinical data, such as eXtasy, Exomizer, PhenoVar, Phen-Gen, and Phevor (Sifrim et al. 2013; Javed et al. 2014; Robinson et al. 2014; Singleton et al. 2014; Trakadis et al. 2014). There are several possible outcomes of a WES or whole genome sequencing (WGS) approach (Fig. 1): 1) detection of a known disease causing variant in a disease gene associated with a clinical phenotype that is the same as that of the patient under investigation, 2) detection of an unknown variant in a known disease gene with a matching clinical phenotype, 3) detection of a known variant in a known disease gene with a non-matching phenotype, 4) detection of an unknown variant in a known disease gene with a non-matching phenotype, 5) detection of an unknown variant in a gene not previously associated with disease, and 6) no genetic variant detected that could explain the phenotype of the patient. There are several other possible outcomes, but those described above are the most frequently encountered results, in our experience. Strictly speaking, only in the case of the first outcome, a genetic diagnosis has been reached with certainty. In the second outcome, if the allele frequency in the normal population is very low to zero, and the variant results in a change of a splice site or a missense mutation at a highly conserved position in the gene, or a stop- or frame shift mutation in genes in which such null-mutations have been shown to be pathogenic, in most cases the result will be considered a conclusive diagnosis. Although one should be careful in the case such a diagnosis is partially based on computational pathogenicity predictions. In the third, fourth, and fifth outcome described above, a certain diagnosis has not (yet) been reached. For the evaluation of variants in scenarios 2–5, there are several additional genetic factors that should be taken into consideration, for example segregation data, the type of mutation (e.g., nonsense, frame shift, de novo, and so on), prevalence data, phylogenetic data, chemical properties of changed amino acids, and computational pathogenicity predictions. This has been described in more detail in a paper on the recommendations by the American College of Medical Genetics and Genomics and the Association for Molecular Pathology about the standards and guidelines for interpretation of genetic variants (Richards et al. 2015). In these guidelines, there are five criteria regarded as strong indicators of pathogenicity of unknown genetic variants. These are: (1) the prevalence of the variant in affected individuals is statistically higher than in controls, (2) a variant results in an amino acid change at the same position as an established pathogenic variant, (3) a null variant in a gene where loss-of-function is a known mechanism of disease, (4) a de novo variant, with established paternity and maternity, (5) established functional studies show a deleterious effect. In the case of IEM in general, de novo mutations appear not to be the major disease causing mechanism. Nevertheless, there are many IEM associated genes in which de novo mutations are a rare finding, and, more importantly, in a small number of genes de novo mutations are encountered frequently, for example *ABCD1* in which mutations cause X-linked adrenoleukodystrophy (Wang et al. 2011), *SLC25A4* causing an early-onset mitochondrial depletion syndrome (Thompson et al. 2016), and *SLC25A24* encoding a mitochondrial ATP-Mg/phosphate in which de novo mutations cause a progeroid disorder (Writzl et al. 2017). There are currently many computational tools that can be used to predict the effect of genetic variants on the gene/protein function (Cooper and Shendure 2011). Many of these tools, however, have not been designed to be used as a tool to predict pathogenicity in a clinical setting. Moreover, for some of these tools, especially those that combine several individual tools, the training dataset of genetic variants overlaps with the data that were used to evaluate the effectiveness of the tools, potentially causing a bias toward certain predictions (Grimm et al. 2015). For these and other reasons, the output that these computational tools provide should be interpreted cautiously, and should not be regarded as definite proof of pathogenicity. Another factor that is important to note is that in spite of uniform guidelines for the assessment of genetic variants, significant interlaboratory differences in variant interpretation have been observed, especially in the interpretation of “likely benign” and “likely pathogenic” variants (Amendola et al. 2016). For all the reasons mentioned above, and coming back to the criteria described by Richards et al., in many cases functional tests are the only option to obtain conclusive evidence for pathogenicity of variants identified in patients. In some cases, functional data of genetic variants may already be available, for example in databases with prospectively collected functional data of artificially created gene variants. In many cases, this information will not be available, and further investigations of the genetic variant in the patient are necessary. There are different approaches to perform such functional validation studies, and the most frequently applied ones in the field of IEM are described below and in Fig. 2.

## Evidence of pathogenicity based on omics strategies/biomarker studies

Holistic screening approaches can be powerful, broadly applicable tools to obtain additional evidence for pathogenicity of genetic variants. Recently, it has been demonstrated in two independent studies that mRNA expression analysis by RNA-seq can provide important data for pathogenicity of genetic variants that result in changes in mRNA expression levels, e.g., variants causing alternative splice events or causing loss of expression of alleles (Cummings et al. 2017; Kremer et al. 2017). In the study by Kremer et al., combining the results of mRNA expression profile analysis in mitochondrial disease patient fibroblasts with WES increased the diagnostic yield of WES by 10%, in comparison to WES only. In the study by Cummings et al., muscle RNA expression profiles of a patient cohort consisting of primary muscle disorders were analyzed, resulting in a 35% success rate. Whether such a high percentage can also be established for mitochondrial disorder or other IEM with muscle involvement remains to be established.

For IEM, it seems likely that the combination of WES/WGS with metabolomics can provide additional evidence for pathogenicity of unknown variants. In theory, metabolomics should be more informative than mRNA expression profiling, as the metabolome is based on the function of the gene products and not just on the expression of the mRNAs. This is illustrated by the results of an early study in which genetic data was combined with targeted metabolomics data in a genome-wide association study (GWAS), showing that metabolite profiles from sera more than doubled the power of the GWAS, and identified variants in several genes involved in lipid/fatty acid metabolism (*FADS1*, *LIPC*, *ACADS*, *ACADM*) (Gieger et al. 2008). In a large study of nearly 8000 individuals, a similar GWAS approach combining genetic and untargeted metabolomics data resulted in the identification of 145 genetic loci associated with more than 400 plasma metabolites (Shin et al. 2014). In a more recent study, WGS combined with targeted metabolomics identified 101 genetic loci that were associated with plasma levels of 246 metabolites, including variants in 13 genes that are associated with IEM and other genetic disorders (Long et al. 2017). However, there are only a few studies in which WES/WGS and untargeted metabolomics have been combined to diagnose IEM. One of these is a study in which untargeted metabolomics was performed on plasma and urine of a patient with severe developmental delay and skeletal dysplasia. This led to the identification of elevated levels of N-acetyl-D-mannosamine in plasma and urine of a patient, which is the substrate for N-acetylneuraminic acid synthase, also known as sialic acid synthase (van Karnebeek et al. 2016). Exome sequencing performed in this patient revealed variants in *NANS*. This illustrates how metabolomics can be used as a functional complement of genetic data collected by WES to diagnose IEM patients.

Protein expression studies have already proven their value to demonstrate pathogenicity of genetic variants in numerous cases. Usually, protein expression analysis is performed by a simple Western blot in order to demonstrate that genetic variants result in loss of expression of the corresponding protein. However, there are only a few examples of untargeted proteomics studies for the purpose of validating genetic variants. A recent example is a study on defects in *TMEM126B*, a gene encoding an assembly chaperone of the mitochondrial respiratory chain complex I (Heide et al. 2012). In two simultaneously published papers describing the identification of mutations in *TMEM126B* in patients with mitochondrial respiratory chain enzyme complex I deficiency, untargeted protein complexome profiling was used to functionally validate the genetic variants. Complexome profiling is a technique by which native protein complexes are separated by blue native gel electrophoresis, followed by mass spectrometric analysis of tryptic peptides derived from the proteins in the separated protein complexes. By protein correlation profiling of the data, this approach provides detailed information about the composition of protein complexes (Wessels et al. 2009). In the two studies on *TMEM126B* mutations, complexome profiling demonstrated that the variants in *TMEM126B* cause a specific defect in the assembly of the mitochondrial multi-subunit enzyme complex I (Alston et al. 2016; Sanchez-Caballero et al. 2016). These are examples of holistic approaches, that are either untargeted or have a broad scope, which have provided functional data serving as evidence for the pathogenicity of genetic variants.

## Validation of genetic variants by targeted functional assays

In addition to the abovementioned untargeted “omics” approached, there are numerous ways to obtain evidence for pathogenicity of genetic variants by more specific functional assays. For example, in the first report on mutations in the mitochondrial glutamate/H+ transporter GC1 encoded by *SLC25A22*, it was shown that glutamate oxidation in patient-derived fibroblasts was impaired (Molinari et al. 2005). In such experiments, there are in general two ways of establishing a genotype-phenotype correlation as evidence for pathogenicity of genetic variants. The first strategy is to determine if the wild type version of the gene in which the variant has been identified results in a rescue of the phenotype. Usually, patient derived cells are used in such a rescue experiment, e.g., lymphoblasts or skin fibroblasts. The second strategy is to test the genetic variants in model systems. These do not necessarily have to be patient-derived models, but can for example be cell culture models or animal models.

## Rescue experiments

Rescuing of a phenotype can be done by introducing the wild type version of the gene of interest into a cell type, either patient-derived cells with the genetic defect, or a knock-out/knock down model system in which the variant gene is overexpressed. The most important prerequisite for this approach is that the patient cells or model systems show a measurable biochemical or cell biological phenotype that is known to be dependent on the gene of interest. There are several ways to introduce DNA into cells. The selection of the most suitable technique depends amongst others on the cell type and on the functional read-out. For assays in which single cells are tested, e.g., microscopy-based assays, a transient transfection procedure can be applied. For this purpose, the gene, usually a cDNA cloned into a suitable expression vector, is introduced into cells by using an agent or a device that facilitates the uptake of DNA by the cells. One of the oldest methods of transfecting cultured cells is by a calcium-phosphate co-precipitation technique (Graham and van der Eb 1973). Nowadays, there are other, more efficient ways to introduce DNA into cells transiently, for example methods that make use of cationic lipids or by means of electroporation (Neumann et al. 1982; Felgner et al. 1987). The efficiency of these relatively simple and quick transient transfection procedures is often (well) below 100% and is very much cell type dependent, making it potentially less suitable for subsequent measurements in cell populations, e.g., enzyme measurements in transiently transfected cell cultures, as the transfected cells are diluted by non-transfected cells in the same cell culture. However, for assays in which individual cells are examined, such as microscopic assays, it can be a very suitable technique. This has for example been demonstrated for Zellweger-spectrum disorders, where transfection by electroporation of patient-derived cultured fibroblasts with a combination of a wild type peroxisomal gene combined with a peroxisomally targeted green fluorescence protein (GFP) was performed. Rescue of the phenotype was scored by microscopic examination of peroxisomal targeting of GFP in the transfected cells (Ebberink et al. 2010).

For functional assays that require near 100% transfection efficiency, a transient transfection procedure could be used when followed by a cell sorting step, e.g., by including a gene encoding green fluorescent protein in the transfection procedure and sort fluorescent cells by FACS. Another more frequently used approach is to use viral vectors in combination with antibiotics resistance markers, that make it possible to reach a 100% transduction efficiency. A lentiviral transduction system is often used for this purpose, as it is safe and allows for efficient transduction of almost all cell types, including non-dividing primary cells (Naldini et al. 1996). Lentiviral transduction of patient-derived fibroblasts is a powerful procedure to prove that a functional defect in patient-derived cells can be rescued by the wild type version of a cDNA of the gene of interest, provided that the cells show a functional/biochemical defect. In the case of mitochondrial disorders, such a defect could be a deficiency of one or more respiratory chain enzymes, which can be measured by spectrophotometric methods (Rodenburg 2011). Alternatively, respirometry has been used as a more broadly applicable read-out for lentiviral complementation of mitochondrial defects (Kremer and Prokisch 2017), although it is less sensitive and less specific than individual respiratory chain enzyme measurements. Lentiviral transduction of patient fibroblasts has been described in studies of all oxidative phosphorylation complexes, as illustrated by these recent examples that include complex I: *NDUFA9* (Baertling et al. 2017), complex II: *SDHA* (Renkema et al. 2015), complex III: *LYRM7* (Hempel et al. 2017), complex IV: *PET117* (Renkema et al. 2017), and complex V: *ATP5A1* (Jonckheere et al. 2013). Also for combined enzyme deficiencies, e.g., caused by defects in mitochondrial translation, lentiviral transduction has been shown to be of value in functional genetics studies, for example *TRMT10C* (Metodiev et al. 2016), *TSFM* (Emperador et al. 2016), and *PNPT1* (Alodaib et al. 2016). These are just a few of many examples that have been published in the field of mitochondrial disorders in recent years. Lentiviral transduction experiments have also been applied to other IEM. An example of this is the investigation of fibroblasts from patients with 2-aminoadipic 2-oxoadipic aciduria, caused by mutations in *DHTKD1*, that were shown to accumulate 2-oxoadipate. This could be normalized by expression of wild type *DHTKD1* cDNA in these patient-derived cells (Danhauser et al. 2012). It is important to note that there are also pitfalls to the use of genetic complementation experiments. After transduction, the expression of the cDNA is under the control of the transcriptional regulatory elements present in the lentiviral vector, which may lead to different expression levels in comparison to the endogenous wild type gene of interest in control cells. As patient cells can in some cases also be growth-compromised because of the genetic defect, this may result in accelerated cell death of transduced cells (Wanschers et al. 2014). In addition, in cases in which the mutant gene is still expressed, there could be a competition between the endogenous and exogenous gene product, which may result in unexpected results. Therefore, it is important to include proper control experiments when doing lentiviral transduction studies. These controls should include a negative control for the transduction procedure. An option could be to include a transduction of the mutated cDNA in patient-derived cells as an additional negative control. However, as many mutant alleles are expressed at reduced levels in patient cells, the possibility that exogenously overexpressed mutant alleles may result in artifacts should be taken into account. Therefore, one should be very careful with the interpretation of the results of these mutant allele expression studies, as also explained further below.

## Transgenic expression in model systems

As an alternative to the rescue experiments described above, expression studies of genes containing variants of unknown clinical significance in a model system, followed by suitable functional assays, is often performed to investigate the functional consequences of genetic variants. Such experiments can provide strong evidence for a specific genetic variant to impair the function of the encoded protein. Whether or not this could be a suitable approach should be considered carefully, especially in the case the variant protein is not expressed in the patient. Often, pathogenic mutations result in reduced protein expression levels, also in the case of missense variants. This could for example be due to reduced stability of such a variant protein. The question is what would happen if such a variant protein, that is no longer expressed in the patient, is tested by overexpression studies in a model system. Usually, overexpression experiments include a test for the expression of the mutant protein, before functional studies are performed. However, in the case of a mutation that only affects protein stability but not its function, it is likely that there may be detectable protein levels in such an overexpression study design. This protein, when expressed, could show a considerable residual activity in the functional assay, which may lead to the wrong conclusion that the variant is not pathogenic. Therefore, in cases where it has been shown that the variant protein is no longer expressed in patient tissue samples or in patient-derived cells, one should be very careful with drawing conclusions from studies in which variant proteins are overexpressed. On the other hand, in the case the mutant protein has been shown to still be expressed in the patient, functional studies of the variant protein does provide very specific information about the functional consequences of the mutation. Expression studies can be performed in many different model systems, for example zebrafish, mouse, Drosophila, yeast, or *E. coli*. Some frequently used examples are discussed below.

## Yeast

One of the organisms that is frequently used in functional genetics studies is baker’s yeast (*S. cerevisiae*). It is easy to grow and to genetically manipulate, and genetic knock outs of virtually every yeast gene are readily available. However, yeast can be used for functional analysis of genetic variants in only a subset of human genes. It has been estimated that around 25% of the known human disease genes have an annotated yeast ortholog, and an additional 20% have a yeast paralog (Yang et al. 2017). In addition, not all human gene functions can be tested in yeast, in particular the functions that are specific for higher order organisms. Nevertheless, it is a widely used model organism in functional genetics studies, and some examples of its successful application to IEM are given below. Yeast studies in the field of mitochondrial genetics have a long history (Tzagoloff and Myers 1986). Mitochondrial yeast studies often make use of the fact that respiration deficient strains show poor growth on non-fermentable carbon sources, such as ethanol or glycerol, whereas growth on fermentable carbon sources (e.g., glucose) is normal. The first yeast genes responsible for this phenotype were identified in the early 1960s (Sherman 1963). In a more recent study in which a genome-wide screen was performed to identify genes involved in mitochondrial respiration, more than 10,000 gene deletion strains were tested for differential growth on fermentable and non-fermentable carbon sources. This resulted in the identification of 466 genes essential for respiration (Steinmetz et al. 2002). The human orthologs of many of those genes are now known to be associated with mitochondrial disease. One of the genes identified in the study by Steinmetz et al. as essential for respiration is *MDH2* (in yeast called *MDH1*), encoding mitochondrial malate dehydrogenase. This gene was recently identified as a mitochondrial disease gene (Ait-El-Mkadem et al. 2017). In this study, mutations identified in patients were functionally validated by making use of a yeast *mdh1Δ* strain. The results showed that human *MDH2* carrying the patients mutations failed to rescue growth of the *mdh1Δ* strain on a non-fermentable carbon source, whereas the wild type human *MDH2* cDNA did restore growth of this yeast strain. In addition to mitochondrial disorders, yeast has been used for the functional studies of many other IEMs. One of the earliest examples of this was a study in which a genetic variant in *GALT* encoding galactose-1-phosphate uridyltransferase was investigated. While a cDNA encoding human *GALT* was able to restore the growth on galactose-containing medium of a yeast *GAL7* (the yeast ortholog of human *GALT*) knock out strain, a *GALT* cDNA with the patients mutation was unable to do so, demonstrating that the amino acid substitution disrupts *GALT* function (Fridovich-Keil and Jinks-Robertson 1993).

## Zebrafish

A vertebrate model system that is frequently used to test genetic variants is the zebrafish. This animal is very suitable to study (amongst others) developmental abnormalities, as for example illustrated by a recent study of classic galactosemia in a zebrafish model showing reduced motor activity and impaired fertility (Vanoevelen et al. 2017). Zebrafish embryos can be genetically manipulated by different techniques. There is a large number of mutant alleles available for further investigations (Kettleborough et al. 2013). One of the most frequently used approaches is the injection of zebrafish embryos with specific morpholinos that block the expression of the gene of interest, after which a capped mRNA carrying the mutation (or wild type mRNA as control) can be injected and the phenotype of the developing fish can be monitored. An example of a successful application of this approach is a study in which the effects on mutations in *CLPB* on the developing central nervous system were studied to validate the functional consequences of genetic variants in this gene (Wortmann et al. 2015, 2015). *CLPB* encodes a mitochondrial molecular chaperone and is a member of the AAA+ superfamily of ATPases. It is an ATP-dependent disaggregase that solubilizes protein aggregates, and is essential for cell survival during severe stress (Doyle and Wickner 2009). Patients with mutations in this gene show cataracts, neutropenia, and 3-methylglutaconic aciduria, as well as a broad spectrum of neurological involvement with, in the most severe cases, an almost complete absence of neurological development (Pronicka et al. 2017). In the study in which mutations in this gene were described for the first time, all patients showed a neurological phenotype ranging from intellectual disability to congenital encephalopathy with progressive brain atrophy (Wortmann et al. 2015, 2015). In the zebrafish model, injection of CLPB morpholinos induced clear signs of cerebellar abnormalities that could be rescued by wild type human *CLPB* mRNA but not by *CLPB* mRNAs carrying the patient mutations, demonstrating a causal relationship between the neurological phenotype and the *CLPB* mutations. Another example is the study of a genetic variant in *DGAT2* (encoding diacylglycerol acyltransferase 2), that had been identified by exome sequencing in a family with autosomal dominant Charcot-Marie-Tooth disease. In this study, it was shown that the missense variant inhibited the axonal branching in peripheral neurons of the developing zebrafish, providing important evidence for pathogenicity of the *DGAT2* genetic variant as a cause for Charcot-Marie-Tooth disease type 2 neuropathy (Hong et al. 2016). Both studies illustrate that, in spite of a lack of a specific functional assay, the use of the zebrafish model system made it possible to demonstrate the pathogenicity of individual genetic variants in the two genes that were studied. In addition to these examples of studies using the zebrafish model that were initiated on the basis of genetic findings in patients, it has been suggested to develop the model into a medium to high through put system to study genes on a larger scale (Davis et al. 2014), and some steps in this direction have indeed been made (Varshney et al. 2016).

## iPSC

Many disease genes are expressed in a tissue specific manner and encode proteins that have tissue-specific functions, which may be difficult to study in cultured patient-derived cells, e.g., lymphoblasts or fibroblasts, or in model systems, e.g., yeast. Around ten years ago, it was discovered that somatic cells can be reprogrammed into pluripotent stem cells (iPSCs) (Takahashi and Yamanaka 2006). These iPSCs can be differentiated in a variety of cell types, for example neurons or cardiomyocytes. This has opened up new possibilities to study genetic variants in patient-derived differentiated cells in cell culture (Park et al. 2008; Onder and Daley 2012). When creating iPSCs from patient-derived somatic cells, such as fibroblasts, there is a possibility that the genotype of the patient may affect both the generation of iPSCs as well as the differentiation process of iPSCs (Gibson and Thakkar 2017). For mitochondrial disorders, it has been shown for iPSCs generated from fibroblasts of patients with Leber’s hereditary optic neuropathy (LHON) caused by mtDNA mutations that neither the reprogramming process nor the pluripotency of the generated iPSCs, as determined by a teratoma formation test, were altered (Hung et al. 2016). By contrast, another study has shown that the mtDNA mutation load in iPSCs derived from the so-called mutator mouse, with a proofreading defect in the mtDNA polymerase γ, does affect the differentiation potential of iPSCs (Wahlestedt et al. 2014). Furthermore, inhibition of mitochondrial energy metabolism by rotenone or oligomycin, as well as genetic disruption of the mitochondrial transcription factor Tfam, has been shown to interfere with the differentiation of iPSCs into the neurogenic lineage (Beckervordersandforth et al. 2017). Such effects on iPSCs formation and differentiation may therefore be gene-dependent, or even mutation-dependent. In spite of these issues, iPSCs have been successfully used as a tool in functional genomics studies. In a recent study of coenzyme Q10 deficiency caused by a mutation in *COQ4*, iPSCs were generated from patient fibroblasts (Romero-Moya et al. 2017). The differentiation of the iPSCs into both dopaminergic and motor neurons was shown to be unaltered in comparison to control iPSCs. The patient-derived iPSCs showed clear signs of mitochondrial abnormalities caused by the coenzyme Q10 deficiency. Interestingly, the authors restored the genetic defect by gene editing of the *COQ4* gene by using CRISPR/Cas9, which normalized the biochemical phenotype of the iPSCs. This provided important proof of the pathogenicity of the *COQ4* genetic defect, illustrating the use of both iPSC technology and CRISPR/Cas9 gene editing as tools for the functional validation of genetic defects. Another example in which iPSC technology was successfully applied to investigate the functional consequences of mutations was a study in which iPSC-derived cardiomyocytes of a Barth syndrome patient with a defect in *TAZ* were investigated. The cardiomyocytes showed various functional defects, including irregular sarcomeres, weak contractility, reduced cardiolipin content, and reduced maximal respiration, reproducing some of the cardiac features seen in Barth syndrome patients (Wang et al. 2014). The authors used the iPSC-cardiomyocyte model to test various compounds for potential therapeutic effects, and showed that linoleic acid partially corrected the phenotype of these cells. This study illustrates that iPSCs are not only a useful tool in functional genomics studies, but also can serve as a model system for experiments on therapeutic interventions.

## Databases with functional information

There are several genetic variation databases providing allele frequency about genetic variants (e.g., gnomAD (gnomad.broadinstitute.org), Exome Variant Server (evs.gs.washington.edu/EVS), 1000 Genomes Browser (www.ncbi.nlm.nih.gov/variation/tools/1000genomes), the Leiden Open Variation Database (LOVD; www.lovd.nl), ClinVar (www.ncbi.nlm.nih.gov/clinvar), ClinGen (www.clinicalgenome.org)). The availability of shared genetic data in such databases, as well as the possibility to share information by match-making tools such as GeneMatcher that make it possible to find patients with the same rare condition (Sobreira et al. 2015), are extremely important in the day-to-day practice of clinical genetics. However, there are no such easily accessible and comprehensive databases with functional data of genetic variants (yet). Nevertheless, there are many publically accessible databases containing a wealth of information on gene/protein function that could be helpful in the interpretation of genetic variants. Examples of these are the Kyoto encyclopedia of genes and genomes (KEGG; www.genome.jp/kegg) with information on all cellular functions, including metabolic pathways; Brenda (www.brenda-enzymes.info) with information on enzyme function; and GeneCards (www.genecards.org), with information on all human genes, including links to various other databases. There are also several databases for which a subscription fee is required, for example the Online Metabolic and Molecular Bases of Inherited Disease (ommbid.mhmedical.com). In addition to these general reference databases, there are databases with functional information generated by knock-down or knock-out of entire genes, which does give information on the possible involvement of certain genes in certain pathways. There have been several studies in which genome-wide gene knock-outs (or knock downs) have been performed to obtain functional information, and these data could be of help for the interpretation of WES data. A recent example of this is the study in which a genome-wide CRISPR/Cas9 knock-out screen was performed to identify genes essential for the mitochondrial oxidative phosphorylation system (Arroyo et al. 2016). In this screen, a genome-wide CRISPR single guide RNA (sgRNA) library was transfected into human embryonic kidney cells (HEK293) grown on a glucose-containing cell culture medium, after which a positive selection of transfected cells was performed to remove non-transfected cells. After several days of growth, the culture medium was replaced by galactose-containing medium. Cells that lack a properly functioning OXPHOS do not survive in this medium. The dying cells were harvested by using Annexin V-microbeads, after which the sgRNAs from these dying cells were amplified and sequenced. In this way, 191 genes were identified as essential for OXPHOS, including 72 known disease genes. It should be noted, however, that the screen picked up only a subset of the known OXPHOS genes. This means it is likely that there are many more genes than the 191 identified as essential for OXPHOS in this model system. Nevertheless, the list could in certain cases be informative when interpreting genetic variants identified by WES. As this list is based on functional data, it provides more convincing evidence for a role in mitochondrial OXPHOS than the lists of genes with predicted and partially validated subcellular mitochondrial localization of gene products, such as MitoCarta (Calvo et al. 2016) and Mitominer (Smith and Robinson 2016). However, with respect to the use of such gene lists in WES data filtering, the latter two lists are still very useful as they provide much more comprehensive lists of genes (putatively) involved in mitochondrial functions. There are several other examples of more detailed, prospective functional testing of genetic variants, yielding functional information that could be used in the interpretation of genetic test results. One of the earliest examples of this was a so-called alanine-scan, in which a total of 62 amino acids in three important domains of the hGH-receptor were functionally tested, revealing that at least 12 of the residues are important for hGH-receptor binding (Cunningham and Wells 1989). A more recent example is a study in which more than 600,000 variants introduced in the WW-domain, which is a conserved protein-protein interaction module that is present in many different proteins, were functionally tested for binding by using a phage-display procedure (Fowler et al. 2010). The variants were selected over multiple rounds for binding to peptide coated-beads, and the filtered variants were sequenced, producing a sequence-function map for the WW-domain. More recently, a study was performed in which all possible missence variants in *PPARG* were functionally tested, by expressing a cDNA library with the variants in macrophages lacking endogenous PPARγ, followed by exposure to PPARγ ligand and FACS screening for CD36 expression, which is a target for PPARγ (Majithia et al. 2016). The cDNA sequences in both the CD36+ and CD36- populations were sequenced, and function scores were generated for the different amino acid substitutions. Another very recent example is based on the concept described by Fowler et al., as mentioned above, in which functional maps of missense variants in six human genes (including *TPK1* encoding thiamin pyrophosphokinase) were produced by performing functional tests of variants in a yeast model system in combination with machine learning to refine the functional map of these genes (Weile et al. 2017). Although there are also limitations of these approaches (for example, the availability of a yeast screen) and questions about factors of which the functional consequences may be difficult to test in an overexpression model system (for example mild effects on protein stability), there is enormous potential in such large scale, prospective studies of all known genetic variants, to produce reference databases that could be very helpful in the interpretation of genetic variants detected in patients (Starita et al. 2017).

## Diagnostics or research

When encountering variants in novel disease genes, it is usually possible to obtain functional evidence for pathogenicity through scientific research collaborations, as the results are not only of diagnostic importance, but are also likely to have a scientific impact. However, when investigating unknown variants in known disease genes, the scientific impact of findings may not be as high as in the case of novel disease genes. Nevertheless, for the patient and family members it is very important to obtain convincing evidence of pathogenicity, also in the case of variants in genes that may be less interesting from a scientific point of view. For this reason it is important to implement functional genomics tests in a diagnostic setting. In such a diagnostic setting, the costs and turnaround time of such tests are important issues to take into consideration. In addition, expectations about the possible outcomes of functional tests should be managed by providing information about chances of false positive or false negative test results. For these and other reasons, it is as yet unclear if all of the abovementioned approaches are suitable to be implemented in a diagnostic setting. Acceptable costs and turnaround times are more likely to be achieved when the number of tests reaches sufficient volumes, which is also of importance for maintaining a high quality level and for implementation of proper diagnostic quality control checks. Possibilities to create this are to develop functional assays that are broadly applicable, such as metabolic flux analysis. A higher level of specific functional testing in a diagnostic environment may be achievable when diagnostic centers/functional genomics laboratories focus on specific (groups of) disorders or types of functional tests, and make it possible that other centers can send in diagnostic requests for functional validation studies. Such specialization will also be beneficial for selecting the most optimal approach for functional follow-up of genetic test results. Many types of functional tests depend on the availability of patient material, such as skin fibroblasts or EBV-transformed lymphoblasts, and therefore it is of importance to take the possibility of collecting patient material into consideration when initiating WES/WGS diagnostics.

In conclusion, the introduction of massive parallel sequencing in diagnostic molecular genetics has revolutionized the field of IEM. It has made it possible to identify even the rarest of genetic defects by a routinely performed diagnostic test, such as whole exome sequencing. It has also created new diagnostic challenges because of the detection of increasing numbers of genetic variants of unknown clinical significance. Currently, exome sequencing is operational as a diagnostic test in many genetic centers. The next major step is already taking place, with the introduction of whole genome sequencing in a diagnostic laboratory environment. This will lead to growing numbers of even more challenging questions about the possible functional consequences of unknown genetic variants. Functional tests are a powerful way to demonstrate the pathogenicity of such genetic variants, and therefore the functional genomics laboratory will be an important complement to genetics laboratories in the years to come.
